# Longitudinal cardiovascular magnetic resonance evaluation of progressive pressure overload due to O-ring induced ascending aortic constriction in rats

**DOI:** 10.1016/j.jocmr.2025.101969

**Published:** 2025-10-08

**Authors:** Ida Marie Hauge-Iversen, Einar S. Nordén, Arne Olav Melleby, Lili Zhang, Ivar Sjaastad, Emil K.S. Espe

**Affiliations:** a Institute for Experimental Medical Research, Oslo University Hospital and University of Oslo, Oslo, Norway; b Institute of Basic Medical Sciences, University of Oslo, Oslo, Norway

**Keywords:** Aortic stenosis, Cardiac imaging, Myocardial fibrosis, Translational research, Preclinical research, Diastolic function

## Abstract

**Background:**

Aortic stenosis is a debilitating disease characterized by pressure overload and development of myocardial fibrosis. Animal models that mimic this disease are crucial for translational research. Aortic constriction in rats is commonly used to induce pressure overload, but the precise disease progression in the O-ring induced model of ascending aortic constriction has not been thoroughly evaluated. Additionally, identifying early imaging biomarkers that can predict fibrosis could enhance the model’s translational relevance.

This study aims to evaluate a rat model of progressive pressure overload using cardiovascular magnetic resonance imaging (CMR) by investigating the degree of constriction at different time points and identifying early imaging biomarkers predicting myocardial fibrosis at later stages.

**Methods:**

Sprague Dawley rats (n=14) underwent aortic banding with O-rings (inner diameter of 1.5 mm or 1.3 mm). Sham-operated rats (n=8) served as controls. CMR was performed every fourth week until 20 weeks post-surgery, followed by tissue harvesting and measurements of fibrosis with histology.

**Results:**

All banding groups gradually developed left ventricular (LV) hypertrophy, impaired LV diastolic function (increased E/SRe), increased left atrial (LA) size, and impaired LA function (reduced LA ejection fraction and peak LA strain), but preserved LV ejection fraction during the course of study. The tightest constriction exhibited increased LV fibrosis at 20 weeks. LA diameter at 4 weeks independently predicted LV myocardial fibrosis.

**Conclusion:**

This animal model mimics the gradual progression of stenosis seen in humans, highlighting its translational potential. Early LA diameter predicted myocardial fibrosis. These findings underscore the model’s relevance for studying disease progression in LV pressure overload.

## Graphical abstract

**Figure:**
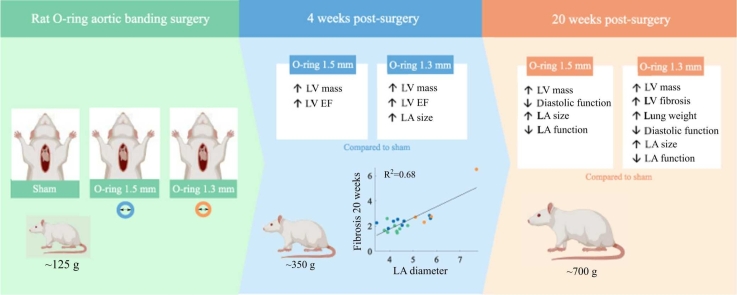
This study evaluated a rat model of progressive pressure overload using O-ring aortic banding with two constriction sizes (1.5 mm and 1.3 mm) in addition to sham-operated controls. Longitudinal magnetic resonance imaging (MRI) assessed the disease development and revealed gradual development of cardiac hypertrophy, impaired left ventricular diastolic function, enlarged left atrial size and reduced left atrial function in the constricted animals. Left atrial diameter at 4 weeks post-surgery was found to be an independent predictor of myocardial fibrosis at later stages.

Plain English Summary/Lay abstract

Aortic stenosis is a condition where the heart has to work harder to pump blood through a narrowed valve. Over time, this extra effort can lead to thickening, stiffening, and scarring of the heart muscle. To better understand how this damage develops, researchers often use animal models that mimic the disease in a controlled way. In this study, we used a rat model to explore how the heart changes when faced with increasing pressure over a long time.

We applied two different levels of narrowing to the main artery (aorta) in rats using small rubber rings. As the rats grew, the narrowing gradually became more severe, similar to what happens in humans with aortic stenosis. We used MRI to follow changes in the heart every four weeks for five months.

We found that the hearts of rats with the tightest narrowing developed more scarring (fibrosis), thickened walls, and signs of impaired function, especially in how the heart relaxes and fills with blood. Importantly, we discovered that an early increase in the size of the left atrium (a small chamber in the heart, where the blood waits before it is let into the main pumping chamber) was a strong predictor of later heart scarring.

This study highlights how advanced imaging techniques, particularly cardiac MRI, can noninvasively track disease progression and detect early signs of pathological remodeling. The model offers a robust platform for testing interventions and for identifying imaging-based biomarkers that may help guide early treatment decisions in patients with pressure overload, such as those with aortic stenosis.

## Introduction

Cardiovascular diseases remain a leading cause of morbidity and mortality worldwide, and conditions resulting in pressure overload, such as aortic stenosis, pose a significant clinical challenge [1], [2], [3]. Currently, the primary treatment for aortic stenosis is surgical or transcatheter aortic valve replacement (TAVR) [4]. In today’s practice, this is typically performed in patients with symptomatic severe aortic stenosis. However, recent evidence suggests that patients in earlier stages of aortic stenosis may also benefit from TAVR [5]. Therefore, translational research is crucial to gain more knowledge about early disease progression and its connection to adverse remodeling and facilitate the optimization of the timing of TAVR.

Rodent models of pressure overload that mimic the effects of aortic stenosis are essential for advancing our mechanistic understanding of disease development. Of the two major models used for this, ascending aortic constriction (AAC) more closely models aortic valve stenosis than transverse aortic constriction (TAC) as the constriction causing the pressure overload is located closer to the aortic valve [6], [7]. In AAC, the proximity of the ring to the LV produces a hemodynamic profile that more closely resembles what is seen in patients with aortic stenosis [8], whereas in TAC a steal phenomenon is relevant through the a. brachiocephalic artery. However, despite their utility, traditional rodent models of pressure overload induced by suture constriction often fall short in replicating the slow progressive nature observed in humans, and often result in severe eccentric left ventricular (LV) remodeling and lung congestion within few weeks [8]. Therefore, developing rodent models that exhibit slow disease progression and controlled development of the degree of aortic constriction over time would be valuable, as they would more accurately capture the clinically relevant pathophysiological processes and mechanisms that are involved in early disease.

Preclinical cardiovascular research primarily employs mice and rats. While mice enable higher throughput and are favored for genetic studies [7], rats are larger and more physiologically similar to humans [9], making them a suitable model for investigating various cardiovascular pathologies due to their increased blood volume and heart size, which facilitate comprehensive blood work and tissue analysis [7].

One major area of interest for understanding disease progression in aortic stenosis is myocardial fibrosis, which has demonstrated prognostic and diagnostic value in heart diseases [10], [11], [12], [13]. Cardiac fibrosis is also found to be a predictor of all-cause mortality in patients with aortic stenosis [14], [15]. Imaging biomarkers capable of predicting myocardial fibrosis would therefore be invaluable for accurately capturing the heart’s progressive remodeling.

Cardiovascular magnetic resonance imaging (CMR) is the established gold standard for assessing cardiac volumes and is well-suited for longitudinal investigation of cardiac structure and function [16], [17], with demonstrated value in the preclinical setting [18], [19]. CMR allows assessment of changes in systolic and diastolic function [20], [21] by measuring cardiac volumes and mass, peak global longitudinal strain (GLS), mitral flow velocities, myocardial deformation and left atrial (LA) size and function [22].

In this study, we aimed to investigate the disease development in a rat model of slowly developing pressure overload by CMR. We sought to map the longitudinal development of the disease under two different degrees of constriction and identify imaging biomarkers early in the disease course that predict myocardial fibrosis at later stages of the disease, demonstrating the rat model’s value as a tool for translational research.

## Methods

### Ethics

All animal experiments followed local guidelines and were approved by the Norwegian National Animal Research Committee (approval #20208), which conforms to the National Institutes of Health guidelines (NIH Publication No. 85–23, Revised 1996).

### Study design

Male Sprague Dawley rats (Janvier Labs, Le Genest-Saint-Isle, France), weighing ∼125 g at the time of surgery, underwent either aortic banding or sham surgery (around 1 month old). The animals were housed 2–3 per cage, maintained on a 12-hour light-dark cycle, and provided with ad libitum access to food and water. CMR was performed starting at 4 weeks after surgery and continued every fourth week until 20 weeks post-surgery for a total of 5 examinations. 20 weeks post-surgery, animals were sacrificed, the heart was excised, and the myocardium was weighed and stored for histology with Masson’s Trichrome staining and droplet digital polymerase chain reaction (ddPCR). All analyses were performed by a single operator blinded to the surgical intervention and the results of other analyses.

### O-ring ascending aortic banding surgery

To induce pressure overload, ascending aortic banding was carried out with spliced rubber O-rings according to a method previously established by our group [23]. Anesthesia was initiated with 4–5% isoflurane in oxygen. Following intubation, the animals were placed on a specialized small-animal ventilator (VentElite, Harvard apparatus, Holliston, Massachusetts), and anesthesia was maintained with 2% isoflurane in oxygen. The thoracic cavity was opened, and an O-ring (Polymax, Bordon, United Kingdom) with an internal diameter of 1.5 mm (n=8) or 1.3 mm (n=6) was sliced open and placed around the ascending aorta. The two ends of the O-ring were then re-ligated with sutures. The choice of inner diameters of the O-rings was based on the survival in a pilot study that included four O-rings with inner diameters ranging from 1.07 to 1.5 mm (Supplemental Table S1). Sham-operated animal (n=8) served as controls and underwent the same surgical procedure without the insertion of an O-ring. Both local and systemic analgesia were provided before surgery. The animals received 0.6 mg of bupivacaine at the incision site prior to the surgery, along with a subcutaneous injection of 0.05 mg/kg buprenorphine administered 30 min before surgery and then 8, 16, and 24 h post-surgery. A total of 28 animals underwent surgery for this study, and all survived for at least 24 h post-surgery, and 22 survived until the end of the study at 20 weeks post-surgery. Persistent stenosis was visually confirmed by echocardiography before harvesting, and the ring was inspected after harvesting.

### CMR protocol

For CMR, anesthesia was induced with ∼4% and maintained with 1.5–2% isoflurane in oxygen. CMR imaging was performed using a 9.4 T magnet (Agilent Technologies, Palo Alto, California) interfaced to an Avance Neo console (Bruker Biospin, Ettlingen, Germany). During the entire examination, electrocardiography, respiration rate and body temperature were monitored. Body temperature was regulated with heated air aiming at maintaining a steady state at 37 °C.

The cine examination included 4- and 2-chamber long-axis view as well as a stack of short-axis slices covering the entire LV and LA. The short-axis stack was obtained with 4x undersampling and was reconstructed using compressed sensing as previously described [24]. The long-axis views were acquired in a fully sampled manner. Key acquisition parameters included: slice thickness 1.5 mm, matrix 128 ×128 for fully sampled images and 128 ×32 for compressed sensing, field of view 45 ×45 mm^2^, repetition time 4.17 ms, echo time 2.12 ms, flip angle 12° for fully sampled and flip angle 15° for compressed sensing. Signal averaging was set at 2–3, and the total acquisition time was approximately 20 min.

For myocardial strain assessment, CMR tissue phase mapping (TPM) was conducted in a 4-chamber long-axis view and in a short-axis midventricular view using a radiofrequency-spoiled black-blood gradient echo nine-point phase-contrast protocol [25]. A compressed sensing technique with 4x undersampling was employed as previously described [24]. Data acquisition was performed in a non-interleaved manner, resulting in a temporal resolution equivalent to the repetition time. Key acquisition parameters for TPM included: slice thickness 1.5 mm, acquisition matrix 128 ×32, field of view 45 ×45 mm^2^, repetition time 4.17 ms, echo time 1.98 ms, velocity encoding strength 20 cm/s, and flip angle 10°. Image frame numbers were set to cover more than one heart cycle. The acquisition time was approximately 4 min per slice.

Phase-contrast CMR was used to measure mitral flow. Three slices were positioned perpendicular to the transmitral blood flow as observed from the two long-axis cine views. The middle slice was placed at the tip of the mitral leaflets during mitral valve opening, and the two additional slices were planned from the middle slice by shifting them ±0.5 mm along the normal vector of the slice. Same as with TPM, a radiofrequency-spoiled gradient echo nine-point phase-contrast protocol with 4x compressed sensing was utilized, but without the black blood preparation, as previously described [26]. Key acquisition parameters for CMR mitral flow included: slice thickness 1 mm, matrix 128 × 32, field of view 45 × 45 mm^2^, repetition time 4.17 ms, echo time 1.98 ms, velocity encoding strength 200 cm/s, and flip angle 10°. Image frame numbers were set to cover more than one heart cycle. The acquisition time was approximately 4 min per slice.

One animal in the 1.3 group lacks CMR data at the first two time points.

### CMR analysis

Data from cine was analysed using Segment v3.2 R8456 (Medviso, Lund, Sweden, segment.heiberg.se) [27]. TPM and mitral flow data were analysed with Matlab R2018 (The MathWorks, Inc., Natick, Massachusetts). Undersampled data underwent compressed sensing reconstruction with a temporal Fourier compressed sensing reconstruction algorithm using Matlab R2018 (The MathWorks, Inc.), as previously described in detail [24].

LV volumes, LV mass and LA volumes (LAV) were measured from the cine short-axis stack. LA ejection fraction (LA EF) was defined as LAV(max)-LAV(min)/LAV(max). LA diameter was measured from the 4-chamber long-axis CINE images at two time points in the cardiac cycle, when the LA was largest (maximum LA diameter) and smallest (minimum LA diameter).

LA strain analysis was carried out on cine images using feature tracking in Segment v3.2 R8456 [27], [28]. The atrial wall was segmented, and LA strain was semi-automatically calculated for each time point in the recording. Peak LA strain was defined as the maximum LA strain achieved during the cardiac cycle. From the LA strain curve, LA strain rate was calculated by taking the derivative. Positive peak LA strain rate was defined as the maximum value on the LA strain rate curve, while negative peak LA strain rate was defined as the most negative value on the LA strain rate curve.

TPM recordings were semi-automatically segmented to obtain LV strain and strain rates, using a previously described software in Matlab R2018 (The MathWorks) [25]. Peak global circumferential strain was defined as the minimum strain in circumferential direction in the midventricular short-axis view. Peak global longitudinal strain was defined as the minimum strain in the longitudinal direction in the 4-chamber long-axis view. SRe(long) was defined as peak positive strain rate in early diastole and SRa(long) the peak positive strain rate in late diastole, and both were measured in the longitudinal direction from the 4-chamber view.

The mitral flow assessment was standardized by choosing the region of interest from the time point of peak velocity in early diastole. This was performed by first drawing a region around the mitral orifice in the initial time frame and plot the velocities as a function of time. From this plot, the time point of peak velocity in early diastole was identified and a new, precise region of interest was drawn around the mitral inflow, only including pixels of the blood flow. The pixel-wise velocities within this region were plotted, and maximum velocities in early and late diastole were identified in all three slices. E and A were defined as the maximum velocity across the mitral valve in the three slices for early and late diastole, respectively. The composite parameters E/SRe(long) and E/A were calculated.

### Measuring aortic diameter

To assess changes in aortic diameter as the animals grew, the aortic diameter was measured in the cine 4-chamber long-axis view in all the sham-operated animals from week 4 to week 20 post-surgery. This data was used to quantitatively evaluate the severity of constriction in the O-ring animals. The percentage of unobstructed aortic area at a specific time point was estimated to be the inner diameter of the O-ring (providing an upper limit of the remaining area) divided by the average aortic area of the sham operated rats at the same time point. Based on this calculation, animals were categorized into three severity grades: mild (37–75%), moderate (25–36%) and severe (<25%).

### Histological preparation and analysis

Midventricular sections of the LV were fixed in 10% formaldehyde, paraffin-embedded and sectioned at 6 µm using a microtome (Epredia HM 355S, Thermo Fisher Scientific Inc., Waltham, Massachusetts). Two sections per heart were stained with Masson’s Trichrome using an automatic staining machine (Ventana Medical Systems, Inc., Tucson, Arizona). High-resolution images were captured using an automated slide scanner system (Axio Scan Z1, Carl Zeiss Microscopy, Munich, Germany). The extent of fibrosis was quantified with the open-source software QuPath version 0.4.3 [29] and the amount of fibrosis was reported as the average of the two slices.

### Digital droplet polymerase chain reaction (ddPCR)

RNA was isolated with RNeasy mini (74106, Qiagen Nordic, Oslo, Norway) and resultant concentration was measured with Nanodrop ND-1000 Spectrophotometer (Thermo Fisher Scientific) . cDNA was created with iScript cDNA Synthesis kit (Bio-Rad Laboratories, Inc., Hercules, California). The level of gene expression was assessed by ddPCR using TaqMan assays (Applied Biosystems, Foster City, California) on ABI PRISM 7900HT Sequence Detection System (Applied Biosystems). Expression of collagen I (*Col1a2*), collagen III (*Col3a1*), natriuretic peptide A (*Nppa*), and natriuretic peptide B (*Nppb*) were analysed using QuantaSoft (Bio-Rad Laboratories). Data were normalized to a reference gene, *Rpl32*, and samples from six sham animals were included on all plates for normalization between plates.

### Statistics

Statistical analysis was performed in Matlab R2018 (The MathWorks)). Results are expressed as median (interquartile range). Medians were compared using Kruskal-Wallis test and were adjusted for multiple comparison using Tukey's honestly significant difference procedure if assessment of three or more groups. p<0.05 was considered statistically significant. Univariate linear regression was performed to investigate correlations between parameters. Residuals for each imaging biomarker at 4 weeks were visually assessed for equal variance by plotting residuals against fitted values and assessed for normal distribution of residuals by QQ-plots (Supplemental Fig. S2). Imaging biomarkers with significant correlation from univariate analysis were included in the multivariate analysis. Stepwise regression was performed by iteratively removing the least significant predictor from multivariate analysis, until only imaging biomarkers with independent predictive value of fibrosis remained.

## Results

### Main characteristics

**Fig. 1:**
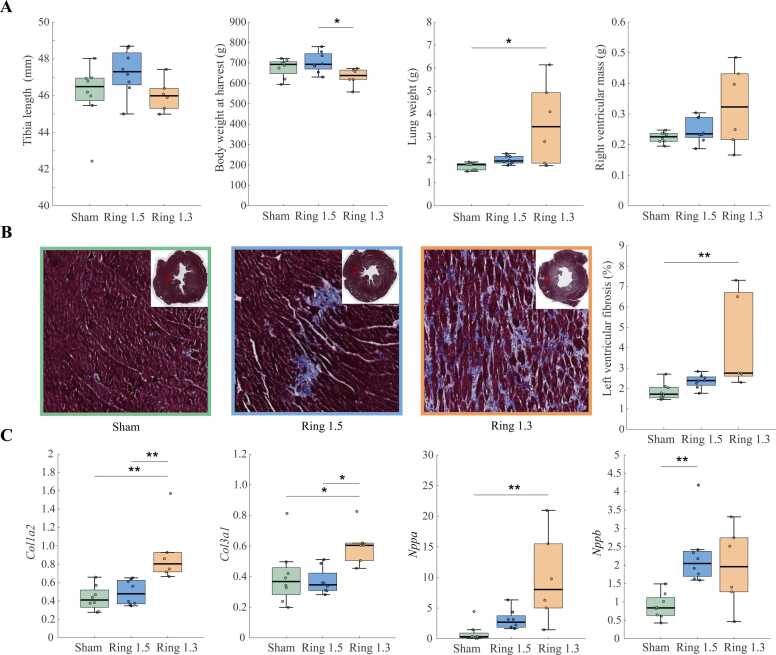
Main characteristics. (A) Box plot of harvest data from 20 weeks post-surgery in O-ring constriction of 1.5 mm, 1.3 mm and sham-operated rats. (B) ***Left:*** Representative images of mid-left ventricular sections stained for collagen with Masson’s Trichrome, 20 weeks post-surgery. ***Right:*** Box plot of median LV fibrosis, 20 weeks post-surgery. (C) Box plot of mRNA levels of collagen I (*Col1a2*), collagen III (*Col3a1*), natriuretic peptide A (*Nppa*) and natriuretic peptide B (*Nppb*). *: p<0.05, **p<0.01.

20 weeks post-surgery, 22 rats (sham: n=8; 1.5 mm group: n=8; 1.3 mm group: n=6) were harvested and similar tibia length was found between all three groups. However, a slightly lower body weight was found in animals in the 1.3 mm group (tightest constriction) compared with the 1.5 mm group (Fig. 1A). Also, the 1.3 mm group had higher lung weight (Fig. 1A and 1C), indicating congestion. RV weight was not different between any of the groups.

### Changes in mRNA expression

Animals in the 1.3 mm group had increased amount of collagen I (*Col1a2*), collagen III (*Col3a1)*, and histology-derived LV fibrosis (Fig. 1B-C). Natriuretic peptide A (*Nppa*) was increased in the 1.3 mm group and natriuretic peptide B (*Nppb*) in the 1.5 mm group compared to sham.

### Progression of aortic stenosis

**Fig. 2:**
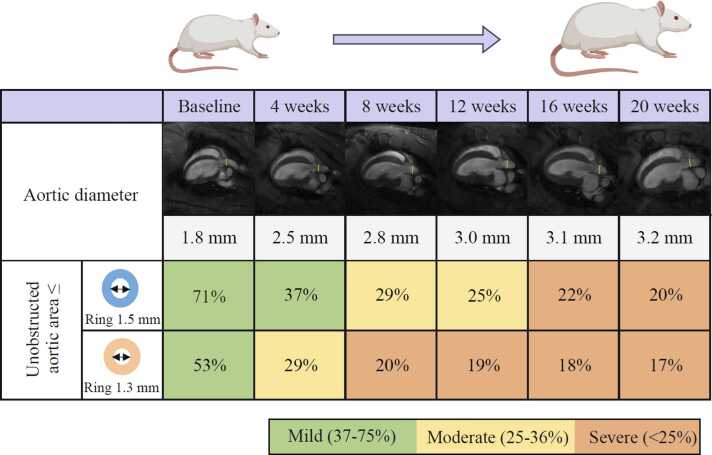
Progression of aortic constriction severity. Aortic diameter increased as the animal grow from the day of surgery (Baseline) until the end of study (20 weeks post-surgery) as illustrated with representative images from long-axis cine CMR. Constriction of the aorta using two different O-rings with inner diameter of 1.5 mm and 1.3 mm, resulting in a gradual decrease in unobstructed aortic area, which will be less than the ratio of the inner area of O-rings to the aortic cross-sectional area. Mild constriction (green) was defined as 37–75% unobstructed aortic area, moderate constriction (yellow) as 25–36% unobstructed aortic area and severe constriction (orange) as less than 25% obstructed aortic area.

The aortic diameter in the animals increased as they grew, with the aorta diameter in the sham group starting at 1.8 mm (1.8–1.8 mm) at baseline to 3.2 mm (3.1–3.3 mm) at 20 weeks post-surgery (Fig. 2 and Supplemental Table S2). No ring was observed to have failed over the course of the study. The gradual increase in aorta diameter corresponded to a more severe relative obstruction. In the 1.3 mm group, the severity of constriction went from a mild constriction at baseline with ≤51% of the aorta area left unobstructed, to moderate constriction at 4 weeks (≤28% unobstructed area) and to severe constriction from week 8 (≤21% unobstructed area). In the 1.5 mm group, the severity of constriction went from mild at baseline with less than 68% of the aorta area left unobstructed, to moderate stenosis from week 8 (≤28% unobstructed area) and to severe stenosis from week 16 (≤24% unobstructed area), demonstrating two different constriction progression rates, both ranging from mild to severe grades.

### LV volumes and mass

**Fig. 3:**
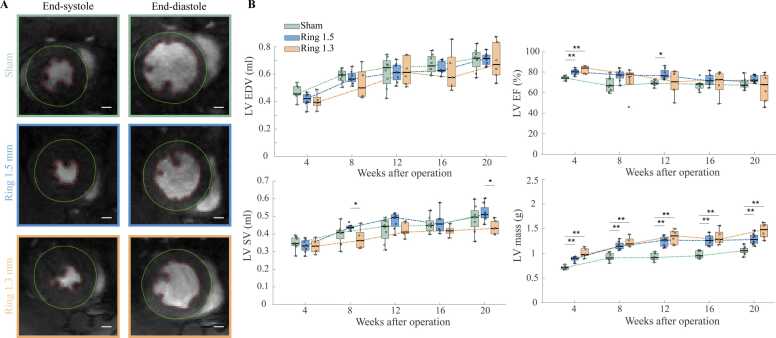
Left ventricular volumes and mass. (A) Representative images of left midventricular short-axis in rats with O-ring constriction 1.5 mm, 1.3 mm and sham-operated rats with epicardial and endocardial trace in end-systole and end-diastole. Scale bar represents 2 mm. (B) Longitudinal development of left ventricular volumes and mass. *EF* ejection fraction, *LV* left ventricular, *SV* stroke volume. *: p<0.05, **p<0.01.

Mean heart rate during the CMR TPM examination for all rats averaged over all time points was 333±43 bpm. LV volumes and mass were examined to investigate the time-dependent LV function and structure. Similar values were found in both banding groups and sham group in the LV end-diastolic volume and LV stroke volume at all time points (Fig. 3). LV EF was increased in both banding groups compared to the sham group at 4 weeks but was similar at all later time points. LV mass was increased in both banding groups at all time points compared to the sham group but were not significantly different between banding groups.

### Mitral flow and LV deformation

**Fig. 4:**
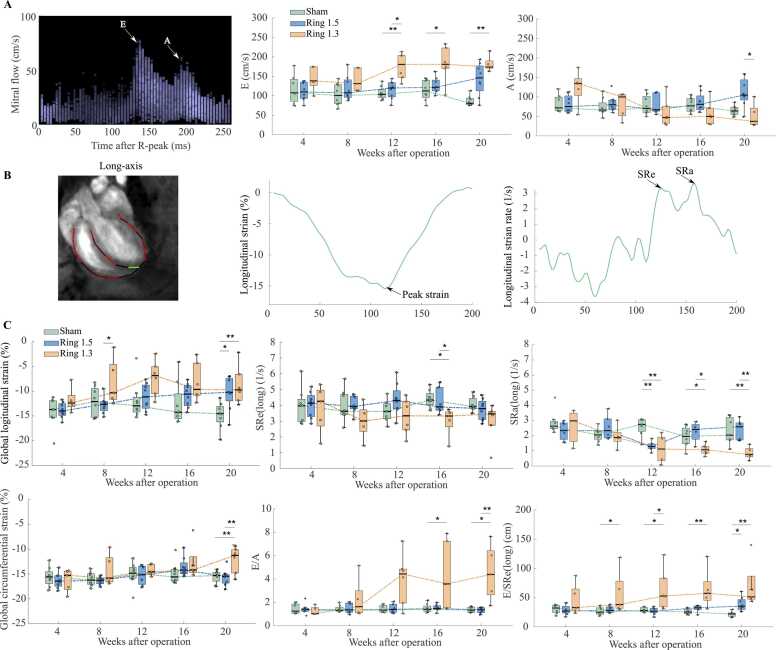
Left ventricular flow and function. (A) *Left:* Representative cardiovascular magnetic resonance (CMR) based mitral flow velocity measurement of a sham operated rat, demonstrating E and A. ***Right:*** Longitudinal box plot of mitral flow measures in O-ring constriction 1.5 mm, 1.3 mm and sham operated rats. (B) ***Left:*** Representative long-axis tissue phase mapping magnitude image of a sham operated rat with longitudinal strain trace. ***Middle:*** Representative curve of longitudinal strain, demonstrating peak strain. ***Right:*** Representative longitudinal strain rate curve, demonstrating SRe(long) and SRa(long). (C) Longitudinal box plots with strain and strain rate, in addition to composite parameter. SR: Strain rate. *: p<0.05, **p<0.01.

To investigate the LV systolic and diastolic function, mitral flow and LV deformation were examined. Peak global longitudinal strain, a parameter of systolic function, was impaired in both banding groups at 20 weeks, and peak global circumferential strain was impaired in the 1.3 mm group at 20 weeks (Fig. 4B-C). SRe(long), a parameter of diastolic function, was found to be reduced in the 1.3 mm group at 16 weeks and SRa(long) was reduced in both banding groups at week 12 and remained reduced in the 1.3 mm group until the end of study (Fig. 4C). Mitral flow recordings were also investigated to assess diastolic function, and E was increased in the 1.3 mm group compared to the sham group from 12 weeks post-surgery until harvest. E/A was increased in the 1.3 mm group from 16 weeks. E/SRe(long) was increased from 8 weeks until the end of study and was also increased in the 1.5 mm group at 20 weeks indicating impaired diastolic function.

### LA size and function

**Fig. 5:**
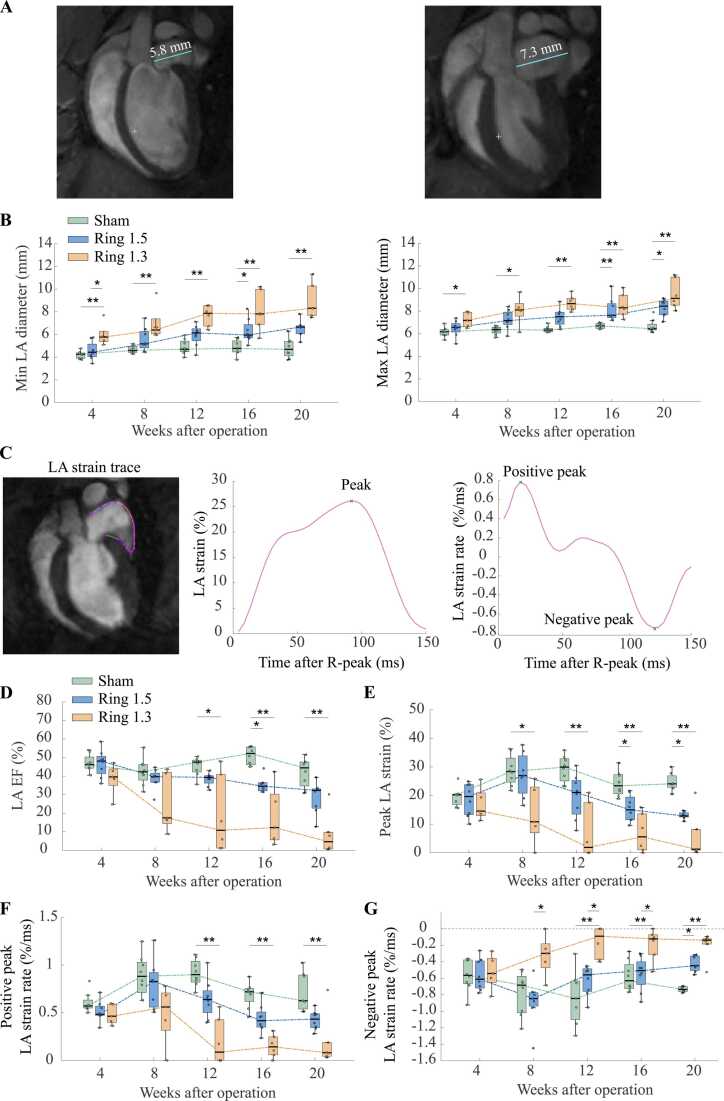
Left atrial size and function. (A) Representative long-axis image of sham operated rat 20 weeks post-surgery with marked trace of ***Left panel:*** Minimum left atrial (LA) diameter and ***Right panel:*** Maximum LA diameter. (B) Longitudinal box plot of LA diameter in O-ring constriction 1.5 mm, 1.3 mm and sham operated rats of ***Left panel:*** Minimum LA diameter and ***Right panel:*** Maximum LA diameter. (C) ***Left panel:*** Representative trace for estimation of LA strain from a long-axis view. ***Middle panel:*** Representative curve of LA strain in a sham operated rat demonstrating peak LA strain. ***Right panel:*** Representative curve of LA strain rate in a sham operated rat demonstrating positive peak LA strain rate and negative peak LA strain rate. (D-G) Longitudinal box plot in O-ring constriction 1.5 mm, 1.3 mm and sham operated rats in the parameters (D) LA ejection fraction, (E) Peak LA strain, (F) Peak positive LA strain rate, and (G) Peak negative LA strain rate. EF: Ejection fraction, LA: Left atrial. *: p<0.05, **p<0.01.

LA size and function were assessed as markers of filling pressure and were found to reflect LV chamber stiffness [21]. Significant increase in LA diameter was found in the 1.3 mm group already at 4 weeks, with changes in both minimum and maximum LA diameter (Fig. 5B). For the 1.5 mm group an increase in LA diameter was found at 16 weeks for both minimum and maximum size. Similar results were also found for LA area and LA volume (Supplemental Fig. S1).

LA function was assessed to gauge alteration in filling pressure. Peak LA strain was significantly reduced in the 1.3 mm group at 8 weeks (Fig. 5E) and in LA EF (Fig. 5D) at 12 weeks. In the 1.5 mm group, LA EF and peak LA strain were both reduced at 16 weeks. Positive peak LA strain rates were significantly reduced in the 1.3 mm group at 12 weeks (Fig. 5F), and negative peak LA strain rate was impaired in the 1.3 mm group compared to the sham group from week 12 and in the 1.5 mm group at week 20 (Fig. 5G).

### Correlation between imaging biomarkers at early time points to fibrosis at 20 weeks

To investigate the relationship between imaging biomarkers at early time points to myocardial fibrosis at 20 weeks, we first performed univariate regression analysis for CMR imaging biomarkers at 4 weeks in addition to ring size. O-ring size, LV mass, peak global longitudinal strain and minimum and maximum LA diameter demonstrated from univariate regression analysis correlation with the amount of fibrosis measured at 20 weeks from histology (Table 1). After stepwise multivariate linear regression, minimum LA diameter was the only imaging biomarkers at 4 weeks with independent predictive value of the amount of fibrosis at 20 weeks. We performed additional analysis where we replaced LA diameter with LA volume and LA area, respectively, and found similar results (Supplemental Table S3 and S4).

**Table 1:** Univariate and multivariate analysis for prediction of fibrosis 20 weeks post-surgery.

	Univariate linear regression			Multivariate linear regression (pre stepwise regression)			Multivariate linear regression (post stepwise regression)		
Imaging biomarkers	β	p-value	R^2^	β	p-value	R^2^	β	p-value	R^2^
Ring size (1.5/1.3)	0.523/2.483	<0.001*	0.45	−0.228/−0.625	0.81	0.76			0.68
LV mass	5.15	0.001*	0.44	2.60	0.44				
LV EDV	0.403	0.93	<0.01						
LV SV	3.29	0.63	0.01						
LV EF	0.0375	0.53	0.02						
E	0.00377	0.45	0.03						
A	0.00647	0.20	0.09						
E/A	−0.138	0.80	<0.01						
GLS	24.2	0.008*	0.33	11.4	0.12				
GCS	5.80	0.67	0.01						
SRe(long)	−0.235	0.32	0.06						
SRa(long)	−0.0548	0.88	<0.01						
E/SRe(long)	0.00988	0.28	0.06						
LA diameter (min)	0.887	<0.001*	0.68	0.607	0.077		0.887	<0.001*	
LA diameter (max)	0.995	0.001*	0.45	0.120	0.75				
Peak LA strain	−0.0772	0.13	0.12						
Positive peak LA SR	−1.16	0.60	0.02						
Negative peak LA SR	1.06	0.43	0.04						

Univariate analysis and multivariate analysis of imaging biomarkers 4 weeks post-surgery in predicting left ventricular (LV) fibrosis at 20 weeks post-surgery. Data represented as the regression coefficient, β, p-value and the coefficient of determination, R^2^.

*EDV* end-diastolic volume, *EF* ejection fraction, *GCS* peak global circumferential strain, *GLS* peak global longitudinal strain, *LA* left atrial, *LV* left ventricular, *SR* strain rate, *SRa(long)* SRa in longitudinal direction, *SRe(long)* SRe in longitudinal direction, *SV* stroke volume. *: p<0.05

## Discussion

This study validates for the first time a rat model of progressive pressure overload using different O-ring sizes to trigger different rates of disease progression. The longitudinal follow-up demonstrates a distinct progression from mild to more severe constriction over time. We observed gradual deterioration of LV diastolic function as well as alterations in LA size and function. LA diameter was identified as an independent predictor of later-stage LV fibrosis, emphasizing its potential for early diagnosis and monitoring of disease progression in experimental animal models. This makes it a valuable tool for gaining new insight into the disease progression in pressure overload and offers an opportunity to explore effects of interventions such as anti-fibrotic treatment, and mechanisms underlying fibrosis development.

### Choice of aortic constriction O-ring size

Whereas the aortic area is dependent on the square of the radius, the resistance to flow is dependent on the radius to the fourth power [30]. Therefore, even a small change in aorta diameter may cause significant changes in the LV afterload. In a pilot study, we tested four different O-ring sizes with inner diameters ranging from 1.07 mm to 1.5 mm. The two tightest O-rings (1.2 mm and 1.07 mm) were excluded from the main study due to high mortality rates (Supplemental Table S1). O-rings with inner diameter of 1.3 mm and 1.5 mm showed good survival (93% and 90% respectively) and were therefore included.

### Growth-related changes in aortic constriction severity

In humans, aortic stenosis progresses gradually from mild to moderate to severe over several years [31], and typically entails gradual loss of function, development of hypertrophy and increased LV chamber stiffness [32]. In contrast, in classical rodent models of aortic constriction the degree of constriction is often fixed. In contrast to the current study employing O-ring-based ascending aortic artery constriction, the majority of previous studies employ transverse aortic constriction (TAC) with sutures, in which the degree of constriction is variable and often not confirmed, and the suture can loosen uncontrolled over time [33]. TAC models in mice commonly use a 27-gauge needle, resulting in an abrupt onset of pressure overload and rapid progression to cardiac dysfunction, contrasting with the gradual progression usually observed in patients [34]. This is in line with our pilot study, which found that starting with a tight constriction that yields an acute moderate or severe stenosis (O-ring 1.2 mm and 1.07 mm, Supplemental Table S1) causes high mortality rates. In contrast, a recent study by Wang et al. [35] investigated the progression of disease over 12 weeks, using a larger, 25-gauge, needle resulting in a milder constriction and demonstrated heart failure characteristics by 8 weeks and severe dilated LV by week 12. This is more similar to human progression, but still there is limited development in constriction severity over time, and the disease developed to heart failure within weeks, indicating a fast disease progression.

Melleby et al. developed a more precise method of aortic constriction in mice using O-rings with fixed inner diameters [23]. In their study they induced O-rings in young mice to improve survival and observed that using O-rings with variable inner diameters resulted in specific cardiac phenotypes over 20 weeks. In this study, we wanted to explore how the growth-related constriction progressed in rats by comparing the unobstructed area at the place of the constriction in the different O-ring sizes at different time points. In this rat study, and in our previous study conducted in mice [23], our goal was to characterize an animal model of aortic valve stenosis by constricting the ascending aorta with O-rings. Both TAC and AAC carry significant merit in investigating pressure overload, and several groups have recently deployed O-rings to achieve TAC in mice [36], [37]. The motivation for using rats in this study was that rats have shown to be closer in physiology and morphology to humans than mice [38] and are larger in size, thereby enabling more tissue for ex vivo and blood work analysis. Consequently, rat models have proven to be of high utility for various heart disease studies [7]. Our results demonstrated progressive and controlled changes ranging from mild to severe constriction over 16 weeks in the 1.5 mm group and over 8 weeks in the 1.3 mm group, while maintaining a slow disease progression, resulting in the absence of LV dilatation even in the tightest constriction after 20 weeks. This shares resemblance with the human disease progression in early stages of aortic stenosis and makes it highly suitable to investigate clinically relevant pathophysiological processes. Using O-rings with different inner diameter also allows studying varying rates of disease progression. This is crucial for translational research, as it mirrors the progression seen in patients, which is believed to significantly impact outcomes [39].

### Translational considerations

Increased afterload in humans has shown to lead to pressure overload, hypertrophy, fibrosis, and diastolic dysfunction [32], and this is also observed in the banded rats in the present study. It is important to acknowledge that humans typically develop aortic stenosis at an old age [40] whereas the animals in this study were young. Humans are thus more likely to have comorbidities and other age-related diseases [41]. The benefit of investigating young animals is a higher survival rate during and after surgery, and a more controlled disease model. In this study, the animals underwent surgery at a young age as a tool for the animals to gradually grow into a more severe aortic constriction. Still, the results must be interpreted with caution, considering the physiological differences between young rodents and elderly human patients, as well as the comorbidities that can influence disease progression and treatment outcomes in humans.

By comparing varying degrees of constriction, we explored how different inner diameters of O-rings impacted the progression of disease over time. We observed that the 1.3 mm group resulted in earlier changes in imaging biomarkers related to both LA and LV diastolic function. These findings emphasize the importance of precise control and awareness of O-ring size choice when designing experimental models to better understand mechanisms underlying disease progression.

### LV systolic and diastolic function

The banding groups had normal LV volumes and ejection fraction throughout the study, except a slight elevation of LV EF at 4 weeks in both banding groups. However, peak global longitudinal and circumferential strain were impaired in both banding groups at 20 weeks post-surgery [42]. This indicates the presence of systolic dysfunction despite preserved ejection fraction, which highlights the importance of careful interpretation of cardiac function assessments based on traditional measurements alone [43]. E/SRe(long) was significantly increased in the 1.3 mm group at eight weeks and in the 1.5 mm group at 20 weeks, and E/A was elevated in the 1.3 mm group from 16 weeks, indicating impaired diastolic function and increased filling pressures in the 1.3 mm group from an earlier time point than the 1.5 mm group. These findings suggest that the degree of aortic constriction directly influences the temporal progression of alterations in diastolic function, with more severe constriction leading to earlier alterations in cardiac filling.

### Evaluating LA size and function

LA diameter was increased in 1.3 mm group already at 4 weeks and in 1.5 mm group at 16 weeks, suggesting alterations in filling pressures. Increased filling pressure is a compensatory mechanism to maintain the stroke volume of the LV and is an indication of later impaired diastolic function [44]. Since the LA wall is much thinner than the LV wall, it is sensitive to changes in luminal pressure [44]. The changes to the LA size therefore suggest a sustained increase in LA pressure over time. Indeed, we found that the LA diameter was one of the earliest imaging biomarkers sensitive to change in both groups compared to the sham group, independent of ring size.

LA size is commonly presented as its maximum size during the cardiac cycle [44]. However, minimum LA size occurs near the end of ventricular diastole and is therefore directly affected by the end-diastolic pressure. It is therefore potentially an even more sensitive marker of abnormal diastolic function than maximum LA size [45]. In this study, both minimum and maximum LA size seems to be sensitive markers of early disease development in this disease model.

The function of the LA can be assessed by either measuring atrial EF, strain or strain rate. We found that peak LA strain was reduced in the 1.3 mm group at 8 weeks, and in the 1.5 mm group from 12 weeks. These results were similar to what we found from LA EF. Our results suggest that LA function may be less sensitive than LA size in this disease model, in contrast to humans where altered LA function is believed to be a more sensitive marker of early changes [22], [46].

### Identifying early predictors of myocardial fibrosis

Concurrent fibrosis and LA dysfunction are predictors for incident heart failure in patients [47], and the development of LV fibrosis has been identified as a crucial target for therapeutic interventions. LV fibrosis is associated with irreversible cardiac remodeling [48], although recent studies indicate that some degree of reversibility is possible [10]. Prediction of myocardial fibrosis is therefore essential for stratifying disease groups to test new therapeutic options and for evaluating the effectiveness of new interventions, both in rodent models [49] and in humans [50].

Since we have a gradual increase of the severity of the constriction, it is possible to investigate in more detail the remodeling over time. We found that LA diameter, LV mass, and peak global longitudinal strain measured at 4 weeks post-surgery were correlated to fibrosis at 20 weeks post-surgery, suggesting that we have cardiac remodeling already at 4 weeks that is connected to the amount of fibrosis seen at a much later time point. LA size emerged as the most reliable predictor. The terminal nature of our fibrosis measurements hampered gauging the temporal development of fibrosis itself, but future studies could investigate the progression of fibrosis over time.

The mRNA expression findings in our study align closely with those reported in our previous study of mice [23], including regarding the increased levels of collagen I and collagen III. Additionally, these results are consistent with earlier studies conducted in rats using aortic constriction [51], [52], [53].

## Limitations

A limitation of this study is the use of only male rats, as the animal model of aortic constriction using O-rings has not yet been established in female rats. It is well-documented that pathophysiology, and consequently imaging biomarkers, can vary between the sexes due to hormonal and physiological differences [54]. Therefore, further studies are necessary to understand how the disease model and imaging biomarkers progress in female rats. It could also be valuable to investigate other species and animal models.

Moreover, in this study, we only had ex vivo tissue for assessment of fibrosis at a single time point. Collecting tissue at multiple time points and investigating how fibrosis develops over time could provide a more comprehensive understanding of the progression of fibrosis and its correlation with imaging biomarkers.

We did not acquire high-resolution CMR cine of the aortic constriction itself in this study. The cine short-axis slices at the level of the O-ring did not consistently capture the lumen profile with the precision needed to quantify the constriction reliably. Future studies could incorporate high-resolution CMR imaging of the constricted aorta to validate the reliability of aortic constriction with O-ring surgery.

No blood pressure measurements were made, so all inferences regarding blood pressure are indirect.

## Conclusions

In summary, this study assesses different rates of disease progression in a rat model of pressure overload using two O-ring diameters. Our longitudinal investigation reveals a transition from mild to severe aortic constriction over time, highlighting significant changes in LV diastolic function and LA size and function. Notably, LA diameter emerged as an early indicator of disease progression and as an independent predictor of LV fibrosis at later stages. These findings underscore the translational potential of this animal model, which effectively mimics the gradual disease progression seen in humans and provide a valuable tool for uncovering disease mechanisms and testing new therapeutic interventions ultimately improving patient care.

## Funding

This study was supported by the KG Jebsen Center for Cardiac Research (Oslo, Norway), The South-Eastern Norway Regional Health Authority (Oslo, Norway) [grant number: 2022013], Familien Blix’ Fond Til Fremme Av Medisinsk Forskning (Oslo, Norway), Olav Raagholt og Gerd Meidel Raagholts stiftelse for forskning (Oslo, Norway).

## CRediT authorship contribution statement

**Ivar Sjaastad:** Writing – review & editing, Validation, Supervision, Resources, Project administration, Methodology, Funding acquisition, Conceptualization. **Emil K. S. Espe:** Writing – review & editing, Validation, Supervision, Software, Project administration, Methodology, Investigation, Funding acquisition, Data curation, Conceptualization. **Lili Zhang:** Writing – review & editing, Supervision, Project administration, Investigation, Conceptualization. **Einar S.Nordén:** Writing – review & editing, Investigation, Conceptualization. **Arne Olav Melleby:** Writing – review & editing, Methodology, Investigation. **Ida Marie Hauge-Iversen:** Writing – original draft, Visualization, Validation, Software, Methodology, Investigation, Formal analysis, Data curation, Conceptualization.

## Declaration of Competing Interest

The authors declare that they have no known competing financial interests or personal relationships that could have appeared to influence the work reported in this paper.

## Footnotes

Appendix A Supplemental data associated with this article can be found in the online version at doi:10.1016/j.jocmr.2025.101969.
