# Supplementary material for: Longitudinal cardiovascular magnetic resonance evaluation of progressive pressure overload due to O-ring induced ascending aortic constriction in rats
Source: J Cardiovasc Magn Reson. 2025 Oct 8;27(2):101969. doi: 10.1016/j.jocmr.2025.101969 (PMC12704282; doi:10.1016/j.jocmr.2025.101969)
Supplement: Supplementary file 1 — Supplemental material [file mmc1.docx]

**Supplementary Results**

**
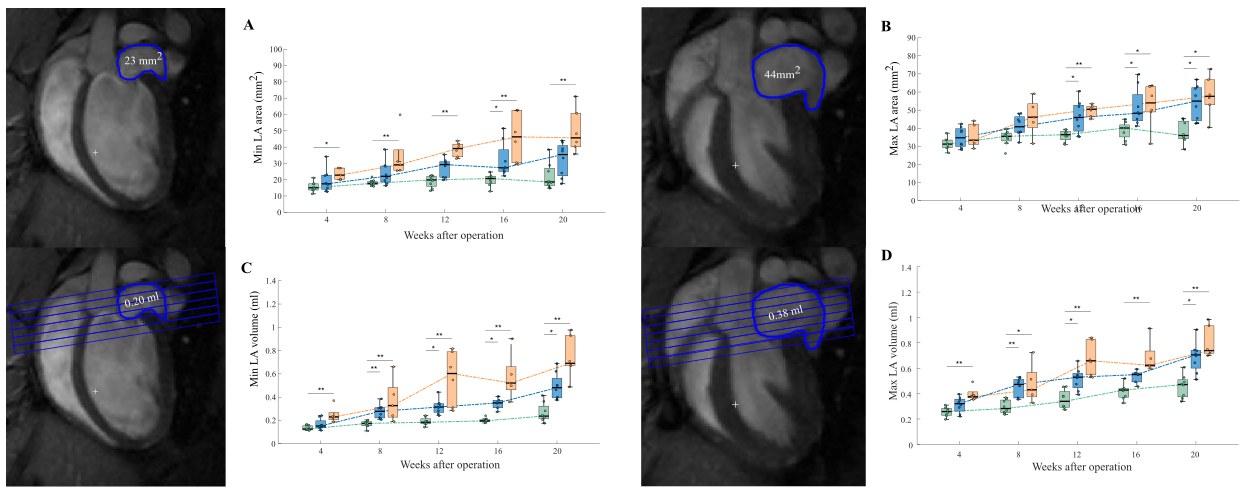
Supplementary Figure S1: *Left panel:*** Representative long-axis image of a control rat 20 weeks post-surgery with marked region/area of interest. ***Right panel:*** Longitudinal box plot left atrial (LA) size measures in O-ring constriction 1.5 mm, 1.3 mm and controls **(A):** Minimum LA area, **(B):** Maximum LA area, **(C):** Minimum LA volume, **(D):** Maximum LA volume. *: p<0.05, **p<0.01

|  | Survive | Dead |
| --- | --- | --- |
| Sham | 7 | 0 |
| Ring 1.5 mm | 9 | 1 |
| Ring 1.3 mm | 14 | 1 |
| Ring 1.2 mm | 6 | 9 |
| Ring 1.07 mm | 7 | 7 |

**Supplementary Table S1:** Results of survival from pilot study. Inclusion criteria was animals that survived the surgery.

| Aortic diameter (mm) | 4 weeks | 8 weeks | 12 weeks | 16 weeks | 20 weeks |
| --- | --- | --- | --- | --- | --- |
| Sham | 2.5 ± 0.5 | 2.8 ± 0.6 | 3.0 ± 0.6 | 3.1 ± 0.7 | 3.2 ± 0.7 |
| Ring 1.5 mm | 2.5 ± 0.1 | 2.8 ± 0.1 | 3.0 ± 0.2 | 3.2 ± 0.2 | 3.4 ± 0.2 |
| Ring 1.3 mm | 2.4 ± 0.1 | 2.8 ± 0.2 | 2.9 ± 0.2 | 3.0 ± 0.1 | 3.2 ± 0.1 |

**Supplementary Table S2:** Aortic diameter (mean ± standard deviation) measured from Cine CMR in sham-operated animals and aortic banded animals with O-rings of inner diameter 1.5 mm and 1.3 mm at given time points after aortic banding surgery. No significant differences were found between the groups at any time point.

|  | Univariate | | | Multivariate  (pre stepwise regression) | | | Multivariate  (post stepwise regression) | | |
| --- | --- | --- | --- | --- | --- | --- | --- | --- | --- |
| Imaging biomarkers | **β** | **p-value** | **R^2^** | **β** | **p-value** | **R^2^** | **β** | **p-value** |  |
| Ring size (1.5/1.3) | 0.523/2.483 | <0.001* | 0.45 | -0.640/-0.921 | 0.95 | 0.73 |  |  |  |
| LV mass | 5.15 | 0.001* | 0.44 | 0.147 | 0.97 |  |  |  |  |
| LV EDV | 0.403 | 0.93 | 0.0005 |  |  |  |  |  |  |
| LV SV | 3.29 | 0.63 | 0.01 |  |  |  |  |  |  |
| LV EF | 0.0375 | 0.53 | 0.02 |  |  |  |  |  |  |
| E | 0.00377 | 0.45 | 0.03 |  |  |  |  |  |  |
| A | 0.00647 | 0.20 | 0.09 |  |  |  |  |  |  |
| E/A | -0.138 | 0.80 | 0.004 |  |  |  |  |  |  |
| GLS | 24.2 | 0.008* | 0.33 | 12.2 | 0.096* |  |  |  |  |
| GCS | 5.80 | 0.67 | 0.01 |  |  |  |  |  |  |
| SRe(long) | -0.235 | 0.32 | 0.06 |  |  |  |  |  |  |
| SRa(long) | -0.0548 | 0.88 | 0.001 |  |  |  |  |  |  |
| E/SRe(long) | 0.00988 | 0.28 | 0.06 |  |  |  |  |  |  |
| LA volume (min) | 13.5 | <0.001* | 0.65 | 14.1 | 0.13 |  | 13.5 | <0.001* |  |
| LA volume (max) | 10.5 | <0.001* | 0.54 | -3.17 | 0.65 |  |  |  |  |
| Peak LA strain | -0.0772 | 0.13 | 0.12 |  |  |  |  |  |  |
| Positive SR peak | -1.16 | 0.60 | 0.02 |  |  |  |  |  |  |
| Negative SR peak | 1.06 | 0.43 | 0.04 |  |  |  |  |  |  |

**Supplementary Table S3:** Univariate analysis and multivariate analysis of imaging biomarkers 4 weeks post-surgery in predicting left ventricular (LV) fibrosis at 20 weeks post-surgery including left atrial (LA) volume. Data represented as the regression coefficient, β, p-value and the coefficient of determination, R^2^. EDV: End-diastolic volume, EF: Ejection fraction, GCS: Peak global circumferential strain, GLS: Peak global longitudinal strain, LA: Left atrial, LV: Left ventricular, SR: Strain rate, SV: Stroke volume. *: p<0.05

|  | Univariate | | | Multivariate  (pre stepwise regression) | | | Multivariate  (post stepwise regression) | | |
| --- | --- | --- | --- | --- | --- | --- | --- | --- | --- |
| Imaging biomarkers | **β** | **p-value** | **R^2^** | **β** | **p-value** | **R^2^** | **β** | **p-value** | **R^2^** |
| Ring size (1.5/1.3) | 0.523/2.483 | <0.001* | 0.45 | -0.655/-0.906 | 0.64 | 0.68 |  |  | 0.60 |
| LV mass | 5.15 | 0.001* | 0.44 | 5.00 | 0.16 |  |  |  |  |
| LV EDV | 0.403 | 0.93 | 0.0005 |  |  |  |  |  |  |
| LV SV | 3.29 | 0.63 | 0.01 |  |  |  |  |  |  |
| LV EF | 0.0375 | 0.53 | 0.02 |  |  |  |  |  |  |
| E | 0.00377 | 0.45 | 0.03 |  |  |  |  |  |  |
| A | 0.00647 | 0.20 | 0.09 |  |  |  |  |  |  |
| E/A | -0.138 | 0.80 | 0.004 |  |  |  |  |  |  |
| GLS | 24.2 | 0.008* | 0.33 | 13.8 | 0.066 |  | 17.6 | 0.017* |  |
| GCS | 5.80 | 0.67 | 0.01 |  |  |  |  |  |  |
| SRe(long) | -0.235 | 0.32 | 0.06 |  |  |  |  |  |  |
| SRa(long) | -0.0548 | 0.88 | 0.001 |  |  |  |  |  |  |
| E/SRe(long) | 0.00988 | 0.28 | 0.06 |  |  |  |  |  |  |
| LA area (min) | 0.110 | 0.001* | 0.44 | 0.0648 | 0.057 |  | 0.0903 | 0.003* |  |
| LA area (max) | 0.0652 | 0.19 | 0.09 |  |  |  |  |  |  |
| Peak LA strain | -0.0772 | 0.13 | 0.12 |  |  |  |  |  |  |
| Positive SR peak | -1.16 | 0.60 | 0.02 |  |  |  |  |  |  |
| Negative SR peak | 1.06 | 0.43 | 0.04 |  |  |  |  |  |  |

**Supplementary Table S4:** Univariate analysis and multivariate analysis of imaging biomarkers 4 weeks post-surgery in predicting left ventricular (LV) fibrosis at 20 weeks post-surgery including left atrial (LA) area. Data represented as the regression coefficient, β, p-value and the coefficient of determination, R^2^.

EDV: End-diastolic volume, EF: Ejection fraction, GCS: Peak global circumferential strain, GLS: Peak global longitudinal strain, LA: Left atrial, LV: Left ventricular, SR: Strain rate, SV: Stroke volume. *: p<0.05

**
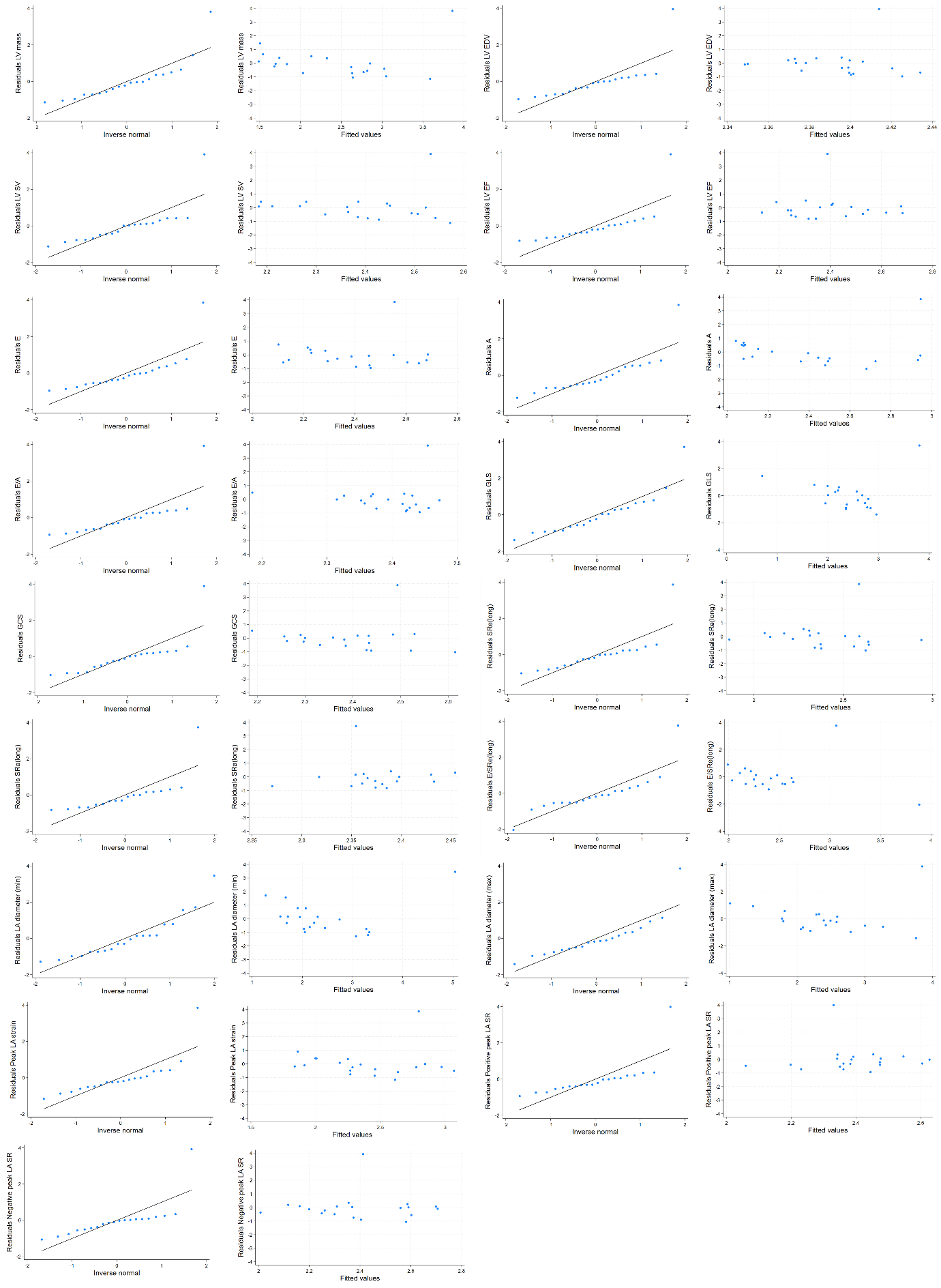
Supplementary Figure S2:** Normal distribution of residuals was assessed using quantile-quantile plots (QQ plots) and variance of residuals against the predicted fitted value was assessed for all imaging biomarkers included in the regression analysis.
